# Comprehensive RNA dataset of AGO2 associated RNAs in Jurkat cells following miR-21 over-expression

**DOI:** 10.1016/j.dib.2016.02.041

**Published:** 2016-02-24

**Authors:** Claudia Carissimi, Teresa Colombo, Gianluca Azzalin, Emanuela Cipolletta, Ilaria Laudadio, Giuseppe Macino, Valerio Fulci

**Affiliations:** Dipartimento di Biotecnologie Cellulari ed Ematologia, “Sapienza” Università di Roma, Italy

**Keywords:** miR-21, AGO2 immunoprecipitation, miRNA target, T-lymphocytes

## Abstract

We set out to identify miR-21 targets in Jurkat cells using a high-throughput biochemical approach (10.1016/j.biochi.2014.09.021[1]). Using a specific monoclonal antibody raised against AGO2, RISC complexes were immunopurified in Jurkat cells over-expressing miR-21 following lentiviral trasduction as well as in Jurkat control cells lines. A parallel immunoprecipitation using isotype-matched rat IgG was performed as a control. AGO2 associated mRNAs were profiled by microarray (GEO: GSE37212). AGO2 bound miRNAs were profiled by RNA-seq.

## Specifications table

**Table:** 

Subject area	*Biology*
More specific subject area	*Molecular biology*
Type of data	Human mRNA microarray; small RNA-seq
How data was acquired	Affymetrix Human Genome HG U133 Plus 2.0 array Illumina Sequencing
Data format	Raw data
Experimental factors	miR-21 was over-expressed in Jurkat cells by transduction with a lentiviral transgenic construct encoding for miR-21 under a PGK promoter. A cognate vector encoding for a control hairpin was used to generate control cell line.
Experimental features	Direct immuno-purification and profiling of AGO2-associated mRNAs in Jurkat cells over-expressing miR-21 using AGO2 monoclonal antibody (11A9, Ascenion) or isotype matched control IgG (Sigma).
Data source location	Rome, Italy
Data accessibility	Data is within this article. mRNA data are available though Gene Expression Omnibus repository (GEO, accession number GEO: GSE37212 ).

## Value of data

• Analysis of purified RISC complexes with associated miRNAs and bound mRNA targets offers an approach to identify functional miRNA targets based on their physical interaction in vivo.
• These data significantly extends the number of bona-fide miR21-target genes
• This dataset could be analysed in conjunction with other AGO2 RNA IP datasets to compare the effectiveness of different techniques to identify new miRNA targets

## 1 Data

The Affymetrix mRNA profile data are provided as CEL files deposited on GEO (GSE37212)(doi:10.1016/j.biochi.2014.09.021 [1]). RNA was extracted from miR-21 over-expressing Jurkat cells (pRRL-21) and matched control cell line (pRRL-Ctrl). For each cell line Input, AGO2 IP and isotype matched IgG IP samples were analysed.

Small RNAs profiled from the same cell lines (AGO2 IP only) are reported as collapsed reads with read counts in tab delimited unix txt format.

## 2 Experimental design, materials and methods

### 2.1 Lentiviral transduction

Viral particles were obtained by co-transfection of 293T-cells with lentiviral plasmid and the PLP-1, PLP-2 and PLP-VSVG plasmids (Invitrogen) and concentrated by ultra-centrifugation. Jurkat cells were transduced at an MOI of 15 and selected with puromycin.

### 2.2 RISC immunopurification

Jurkat cells were lysed in lysis buffer (20 mM Tris–HCl, pH 7.5; 150 mM KCl; 0.5% Nonidet P-40; 2 mM EDTA; 0.5 mM DTT; 1 mM NaF; 40 µ/ml RNasin). Lysates were clarified and pre-cleared by protein-G sepharose beads. An aliquot of total extract was taken out (Input). Monoclonal anti-AGO2 (11A9, Ascenion) and an equal amount of purified rat IgG (SIGMA) were incubated with the pre-cleared lysate. Samples were washed with lysis buffer and wash buffer (50 mM Tris–HCl, pH 7.5; 300 mM NaCl; 5 mM Mg_2_Cl; and 0.05% Nonidet P-40), treated with DNaseI-RNase-free (Promega) and subject to proteinase K digestion. After final wash an aliquot was taken out for western.

### 2.3 mRNA microarray data (Jurkat cells)

mRNAs co-immunoprecipitated (co-IPed RNAs) with anti-AGO2 antibodies from both pRRL21 and pRRL-Ctrl Jurkat cell line were profiled by microarray technology along with total RNAs and co-IPed RNAs with rat IgG. Thus each experimental replica included the following six samples:

**Table:** 

	Jurkat pRRL-21	Jurkat pRRL-Ctrl
Total RNAs	Sample #1 (or *21_Total*)	Sample #2 (or *Ctrl_Total*)
Anti-AGO2 co-IPed RNAs	Sample #3 (or *21_AGO2*)	Sample #4 (or *Ctrl_AGO2*)
IgG co-IPed RNAs	Sample #5 (or *21_IgG*)	Sample #6 (or *Ctrl_IgG*)

We used the Affymetrix Human Genome HG U133 Plus 2.0 array (www.Affymetrix.com) and the Dnavision service provider (http://www.dnavision.com/) to run each microarray experiment and performed it in three biological replicates. However, due to technical failure of one sample (21_IgG) in one replica, only two complete datasets were used for subsequent analyses.

### 2.4 AGO2 bound sRNA profile

sRNAs bound to AGO2 were analysed by Illumina deep sequencing. Identical reads were counted. Data are provided as tab delimited (unix) files. Each line contains two fields:

1. number of reads and
2. read sequence

Adaptor sequences were not removed.

## Funding

This work was supported by the European Commission Framework Program 6 Project “Sirocco” and AIRC (IG-10085) Grants to G.M., by Grants from Associazione Italiana Ricerca sul Cancro (AIRC, IG-10756), CARIPLO Foundation (2009-3603 and 2009-2721).
